# Molecular Cellular Parasitology: The Decades and Challenges Ahead

**DOI:** 10.3389/fpara.2022.963515

**Published:** 2022-07-07

**Authors:** Mark C. Field

**Affiliations:** ^1^School of Life Sciences, University of Dundee, Dundee, United Kingdom; ^2^Biology Centre, Faculty of Science, Institute of Parasitology, Czech Academy of Sciences, University of South Bohemia, Ceské Budějovice, Czechia

**Keywords:** grand challenges, molecular parasitology, cellular parasitology, evolution, genomics, proteomics, therapeutics

## Context

**Figure 1:**
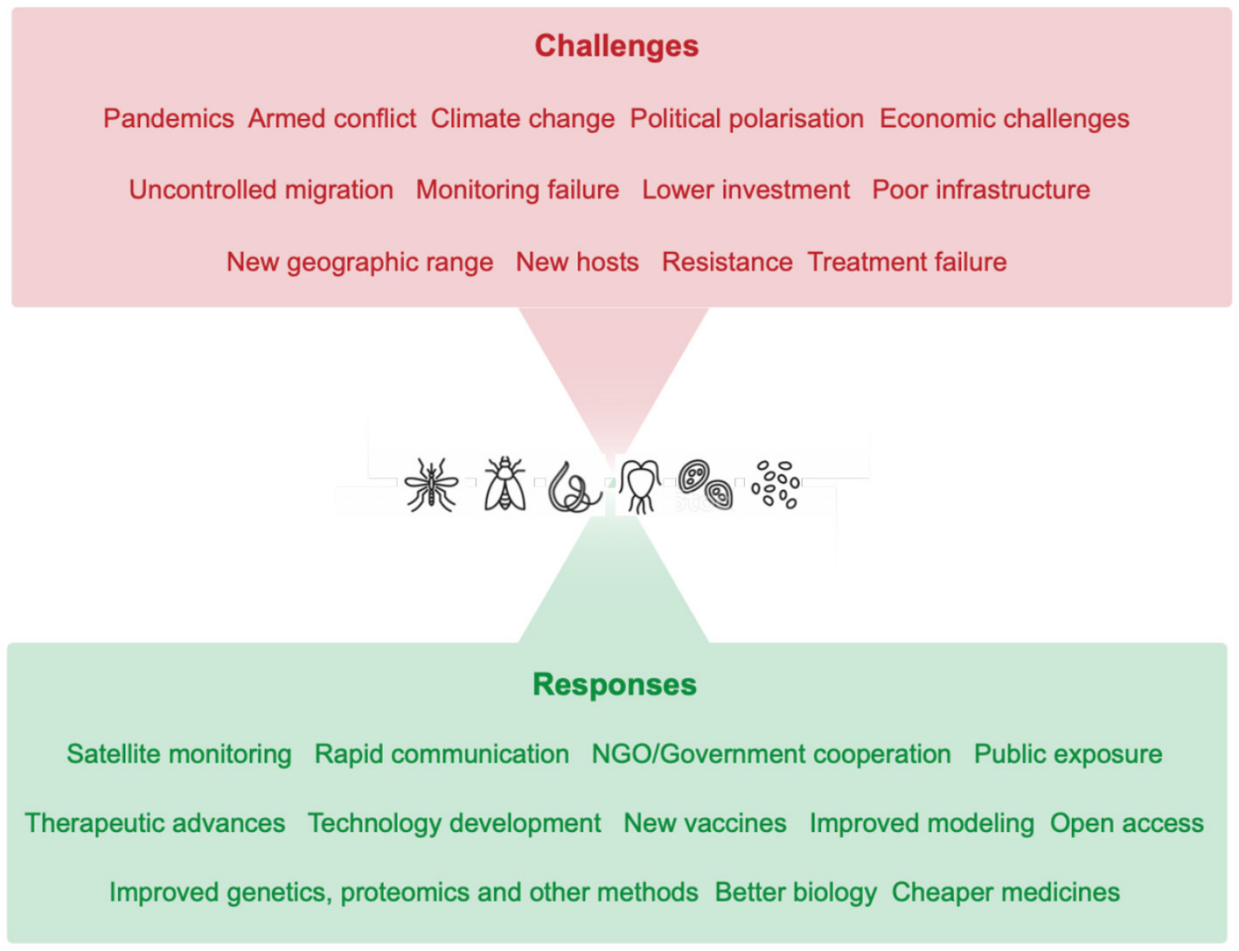
Challenges and responses in managing parasitic diseases. Our world faces multiple challenges (red), many of which act synergistically to destabilize and deflect health care efforts and the quality of life. These factors also have impacts on parasitic diseases, leading to increased cases, morbidity and hardship. In response (green), technological and collaborative approaches promise to negate at least in part these challenges, and many fundamentally rely on molecular and/or cellular-based studies and methods.

Our civilization faces unprecedented challenges, many the result of past inaction (Broecker, 1975). The COVID-19 pandemic drives on in much of the world, there is new war in Europe, continuing conflicts and military persecution of civilians elsewhere and climate change may be close to an irreversible threat to food supply and security, as well as leading to a collapse in biodiversity (Outhwaite and McCann P, 2022). Political extremism and corruption have risen on both the left and the right of the spectrum, carbon-based fuel prices have risen and remain volatile and there is a continual and increasing refugee crisis. We are already witnessing the impacts from these economic, environmental and social challenges toward health, economic prosperity and well-being, with the correlated peril that poverty and disease are intertwined. Altered impacts from diseases caused by eukaryotic pathogens, parasites, are no exception (Figure 1).

Some of these events have direct effects on host and vector ranges. Migrations due to armed conflict, economic and societal concerns and climate change are potentially bringing parasitic infections into naive populations or increasing their prevalence significantly. Other movements, not necessarily classed as migration, can bring parasites directly into an otherwise unaffected population, as has been suggested for *Trypanosoma cruzi* into Europe (Antinori et al., 2017). These events represent challenges to healthcare and the expertise of practitioners now facing new diagnostic challenges.

The news is, however, not all grim. A combination of new or improved technology, innovation and co-operation between NGOs, governments, fundraisers and granting agencies promises much, as evidenced by the London declaration of 2012 (Hotez et al., 2017). Rapid sequencing, diagnostics and cheap computing has put enormously powerful tools into our hands going forward, both for development of therapeutics against parasites and for increasing knowledge of their fundamental biology. I submit that basic biological knowledge is always of huge importance, but when coupled with an understanding of translational potential, can have a direct impact on disease. This is where molecular cellular parasitology has questions, answers and opinions to offer, and I will propose several Grand Challenges which will be complemented by a research topic article collection charging people within the field to discuss those advances and questions that, for them, are the most pressing (Box 1).

Box 1 **Some proposed grand challenges for molecular and cellular parasitology.**

**Accurate life cycles, tissue tropisms and development**

We need to consider more complex life cycles and characterize their importance to pathology and transmission.

**New ‘model' organisms and tools**

Establishment of new tools, including the manipulation of novel parasites.

**Genomics and epigenetics**

Characterizing genome architecture and regulation in 4D, and connect this with disease and development.

**Evolution of immunological resistance, host range and speciation**

Describing relationships between parasite variants, hosts and microbiomes.

**Informatics**

Development of accessible predictive tools with high value where experimental data may be extremely difficult to acquire.

**Genome-wide approaches**

Development of reliable methods to address questions in a genome-wide manner, including inducible expression systems which are lacking for many species.

## Accurate Life Cycles, Tissue Tropisms and Development

Some of the canonical life cycles are coming under strain as evidence mounts for at least additions or more complex interactions than previously considered. For example, we now know that African trypanosomes have considerable presence within the dermal and adipose tissues, and that these sites may participate in mechanisms of transmission, as well as leading to altered sensitivity to therapeutics. For *T. cruzi*, the persistence of small numbers of parasites within the alimentary canal, and not the heart, of the mammalian host was a surprise, indicating that decades old assumptions required revision (Capewell et al., 2016; Ward et al., 2022).

What is also absent from our understanding of these, and many other examples of life cycle transitions, are the signals and transduction pathways that coordinate events, and which are based on robust quantitative data. We have many components and candidates for these processes in hand, but fully integrated understanding remains elusive. Latency in *T. cruzi* disease progression presents an important example here with direct relevance to pathogenesis; currently we have little in the way of details. A recent advance in developing a cell culture model for the blood brain barrier, holds promise for interrogating trypanosome central nervous system (CNS) invasion, and hence molecular level characterization (Speidel et al., 2022), which up until now has been almost entirely phenomenological. The advent of organoid culture, permitting the recreation of three-dimensional differentiated *in vitro* models of organs and tissues, together with applications of microfluidics, may soon permit the molecular level analysis of tropisms, differentiation and development (Ramírez-Flores et al., 2022).

Moreover, mechanisms that mediate tissue tropisms are critically important. The reasons behind restriction of *L. major* to the infection site, contrasted by deep dissemination for *L. donavani*, remain are unclear. Evidence indicates that this is in part due to host immune status, but never-the-less there is a clear difference in the manner in which these species are restricted, with clear clinical relevance. Another example is repression of *Toxoplasma gondii* proliferation from bradyzoite cysts in the CNS, well known to be lost during immunodeficiency (Foster et al., 2022). This Grand Challenge is a call to investigate life cycles; these are challenging and frequently difficult aspects to study and which deserve support as the potential insights we can gain are considerable.

## New “Model” Organisms and Tools

Developing basic toolkits, specifically reliable genetic manipulation and high quality baseline transcriptome and proteome datasets remain to be determined for the vast majority of parasites. These are major deficiencies as in their absence investigation of pathogenesis or any cellular mechanism of interest becomes a challenge. While detailed transcriptome data for *Plasmodium, T. gondii* and several of the kinetoplastids are available, there are deficiencies in the datasets even for the major parasites. These difficulties are frequently technical, for example in obtaining sufficient material; single cell sequencing and amplification methods have, in large part, solved the transcriptome issue but proteomics remains a challenge. At issue are both the difficulty in obtaining sufficient high quality material but also in providing the relevant controls—the field in general lacks a consensus on this, *albeit* that the growth in data itself provides a pathway to improving quality. Insights from ’omics datasets have been highly valuable to assessing *Plasmodium* life cycle and drug interactions, as well as uncovering variations between strains (Rajan et al., 2020). This Grand Challenge then is to provide baseline resources that can further molecular investigations of disease mechanism.

## Genomics and Epigenetics

Understanding how genomes are controlled is central for much biology and has advanced hugely in the last decade or so, but with few exceptions parasitic organisms lag behind. Genome organization and the roles of chromosome-associated domains and other long range interactions between genome components in 4D has expanded our view of the coordination of gene expression, but as the mechanisms for gene expression in many parasitic organisms are non-canonical it becomes critical that these questions are addressed in the specific organisms themselves as inference alone is unreliable. Efforts are ongoing, but the need for progress addressing the influence of environment, drugs, life cycle stage and manipulation of the epigenetic machinery itself remains. Such studies are predicted on the availability of a high quality genome assembly and recent advances in extremely long-read sequencing are valuable here. A related challenge is charting the roles of microRNAs and the many other non-coding RNA molecules, and is in its infancy in protist systems, despite emerging evidence that these elements are critical toward the control of gene expression (Lukeš et al., 2022). The importance here has multiple implications; control of differentiation, the relationship to pathogenesis and uncovering fundamental aspects of control of gene expression, to name a few. Most critical, perhaps, is a comparative analysis across taxa, where specializations and adaptations can be revealed. Overall, this Grand Challenge is to take the understanding of genome architecture and regulation to the next level and connect this with disease.

## Evolution of Immunological Resistance, Host Range and Speciation

Colonizing new host species has obvious selective advantages for parasites, and the transition is well known for many examples. In *T. brucei* we are aware of multiple mechanisms and specific molecules that facilitate evasion of innate host immune defense systems, and which are drivers for entry to new hosts (Dheilly et al., 2019). Host tropisms however are not always clear or well defined, and with several organisms exhibiting broad host ranges, while there are clear groupings of specific parasites into groups, strains, clades, subspecies, distinct typing unit or whichever term is preferred and are likely describing overlapping phenomena. What is clear is that there is considerable flexibility in pathogenesis, drug sensitivity and often host specificity. Related to this is also the role of complex parasite populations, and much as characterizing bacterial microbiomes has been hugely revealing in terms of the complexity and interplay between the microbiome and individuals, it is equally relevant to recognize and understand interactions between parasites and the microbiome, and which has been proposed as a Grand Challenge in itself (Nixon et al., 2020). The potential also for interactions between more than one parasite species infecting a single host are poorly understood. This Grand Challenge is tasked with understanding the relationships between variants, the roles of specific molecules/alleles in defining the species boundaries and the manner in which they interact in the context of protist parasitism.

## Informatics

A major advance in the *in silico* prediction of protein structure was announced in 2021 with the release of alpha-fold and Rosetta, algorithms with significantly superior predictive power from two independent groups. These algorithms have already had considerable impact on life sciences, including parasitology. The ability to obtain an accurate structure prediction, which can facilitate annotation, comparisons and potential functional insights is of considerable power, and can accelerate the discovery process. Full integration of alpha-fold/Rosetta, together with the ability to predict complexes and interaction interfaces is of considerable value. Moreover, integration of such data, together with other predictions or experimental work, has a genuine potential to revolutionize the way on which parasitology can work at the cellular level. Integration of such datasets and the serving of the data back to the community is of considerable complexity, but datasets are available for protein turnover for example, and with intelligent use of web-based elements can bring a huge resource to all. Use of predictive tools is particularly important in some lesser studied or difficult to culture parasites where experimental data may never be acquired and hence accurate predictions are vital. This Grand Challenge has the potential to significantly accelerate and democratize research in terms of bringing sophisticated analysis to most workers in molecular parasitology.

## Genome-Wide Strategies

It is probably highly predictable to offer further development of genome-wide approaches as a Grand Challenge. However, the impacts that genome-wide RNAi methods together with over-expression and CRISPR approaches have had on the field are hard to overstate, and we have seen a massive increase in both the quantity of information being published, together with the quality. These methods have enabled much deeper understanding, rapid assessment of function and the overturning of multiple paradigms, and importantly in a somewhat objective manner. Optimisation of these technologies should permit rapid ’porting of CRISPR/Cas9, for example, to new taxa, with the ability to leapfrog many of the cumbersome approaches used in the past. However, several issues do remain, for example the development of reliable inducible systems and degrons for the direct assessment of essentiality and function of gene products in many parasites.

## Challenging the Worms

So far, I have focused on the protist parasites as examples, but all of the above challenges could equally well apply to infectious worms/flukes. Due to their significantly more complex biology, additional challenges apply to these organisms. For example, in contrast to encouraging developments for protist therapeutics, no new classes of anthelminthics have been approved since the very beginning of the century, and increased coordination and cooperation have been highlighted as important to enable the exploration and development of new treatment modalities (Nixon et al., 2020). This is further complicated by the sophisticated interactions between parasitic worms and the host immune system (Montaño et al., 2021), emphasizing the ongoing need to fully understand life cycles, immune responses and tissue tropisms for these organisms as well as the protist parasites. Roles for extracellular vesicles in mediating some of these aspects of helminth biology are increasingly apparent, but again a challenge here is for the establishment of precise definitions, datasets and approaches (Ryan et al., 2020). Finally in this regard, and perhaps most importantly, is to consider infections in a holistic context; the importance of coinfection has become significantly better appreciated recently, and especially with improved sequence availability for both host and pathogen, for understanding the complex relationships that govern the course of disease. The microbiome can now be defined more easily, and has significant impact for nematode and fluke infections. For example *Neorickettsia spp*. are intracellular bacteria that can infect nematode reproductive tissues, and hence can be transmitted on to the next generation (Formenti et al., 2020), *albeit* that the precise consequences remain to be uncovered. Overall, this Grand Challenge proposes that developing high quality approaches and datasets addressing multiple aspects of parasitic worm infection is critical.

## Conclusions

Many questions have been omitted for obvious reasons of space. These include biophysics, metabolic pathway changes, many features of genome regulation, mapping protein complexes, protein processing and targeting and many more. These are perhaps not all “grand” but represent significant ongoing challenges. There is also a sobering need to retain a sense of realism and relevance. In the face of drug pipelines, where few candidates can ever be expected to progress, the boilerplate “drug target” justification perhaps is less relevant than previously, and tackling this issue is not necessarily the purview of molecular or cellular investigations, but can have a significant impact in competition for funding and other resources. A related question is how to balance a focus on specific organisms with more developed toolkits and understanding with exploring parasite diversity? Technological and conceptual advances mean that molecular studies of parasites will continue apace, but, as argued here, challenges to progress remain. If this section of Frontiers in Parasitology can meet any of these challenges, we will have succeeded.

## Author Contributions

The author confirms being the sole contributor of this work and has approved it for publication.

## Funding

This work was supported by SHEFC through the University of Dundee.

## Conflict of Interest

The author declares that the research was conducted in the absence of any commercial or financial relationships that could be construed as a potential conflict of interest.

## Publisher's Note

All claims expressed in this article are solely those of the authors and do not necessarily represent those of their affiliated organizations, or those of the publisher, the editors and the reviewers. Any product that may be evaluated in this article, or claim that may be made by its manufacturer, is not guaranteed or endorsed by the publisher.
